# Scientific Honors

**Published:** 1892-03

**Authors:** 


### SCIENTIFIC HONORS.

C. W. Stiles, Ph. D., of the Bureau of Animal Industry, the author of the interesting articles, now appearing in the JOURNAL, has been elected corresponding member of the Société de Biologie of France, to fill the vacancy caused by the death of Professor Leidy, of Philadelphia. The Société de Biologie, of France, is one of the most active scientific societies of Europe. The membership is limited to forty active members chosen from France, and forty corresponding members chosen from the world at large, the new members being elected as vacancies occur by death of the former members. The Société de Biologie ranks as the first society in France, next to the French Academy, to which it is considered a stepping stone. Dr. Stiles is at present the only member from the United States. The German members are Professors Virchow, Du Bois Reymond, Von Helmholz, and Leuckart.

